# Semiconductor Quantum Dots: Synthesis, Properties and Applications

**DOI:** 10.3390/nano14221825

**Published:** 2024-11-14

**Authors:** Donghai Feng, Guofeng Zhang, Yang Li

**Affiliations:** 1 State Key Laboratory of Precision Spectroscopy, East China Normal University, Shanghai 200241, China; 2 State Key Laboratory of Quantum Optics and Quantum Optics Devices, Institute of Laser Spectroscopy, Collaborative Innovation Center of Extreme Optics, Shanxi University, Taiyuan 030006, China; 3 Hangzhou Institute for Advanced Study, University of Chinese Academy of Sciences, Hangzhou 310024, China

Semiconductor nanoparticles of sizes smaller than exciton Bohr diameters undergo quantum confinement and are called quantum dots (QDs), which exhibit size-dependent physicochemical properties. For the discovery and synthesis of QDs, three pioneers—Moungi G. Bawendi, Louis E. Brus, and Alexei I. Ekimov—have been awarded the 2023 Nobel Prize in Chemistry [1]. Since the discovery of QDs in the early 1980s [2], the synthesis, properties, and applications of QDs have been extensively investigated [3,4]. Various strategies, including physical, chemical, and biological approaches, have been developed to develop QDs with controllable sizes, compositions, and structures [5,6,7,8]. QDs have superior optoelectronic properties, including wide tunability, narrow emission bandwidth, high brightness, and high efficiency, and offer a wide range of potential device applications in solar energy harvesting [9], lighting [10], displays [11], detectors [12], biomedical imaging [13], and quantum information technology [14].

This Special Issue includes eight contributions, comprising seven research articles and one review article, dedicated to the synthesis, properties, and applications of QDs with diverse components and structures. These studies involve the investigation of the size uniformity in CsPbBr_3_ perovskite QDs via appropriate manganese doping [15], cost-effective magnetic carbon QDs/FeO_x_ photocatalytic composites [16], the effects of surface plasmon coupling on the color conversion from quantum wells into QDs [17], temperature- and size-dependent photoluminescence spectroscopy study on CuInS_2_ QDs [18], methods for obtaining one single Larmor frequency in the coherent spin dynamics of colloidal CdSe and CdS QDs [19], room temperature coherent spin dynamics in CsPbBr_3_ perovskite QDs [20], high-quality CdSe/CdS/ZnS QD-based aptasensors for the simultaneous detection of two different Alzheimer’s disease core biomarkers [21], and a review on advances in solution-processed blue QD light-emitting diodes [22]. Research on the synthesis, properties, and applications of QDs will continue to be rigorous and of high interest. Closely following the prominent event of the 2023 Nobel Prize in Chemistry, our Special Issue highlights the development of QDs and will be of interest to general readers of *Nanomaterials*.
